# Supplementary material for: Complexome profiling of the Chlamydomonas psb28 mutant reveals TEF5 as an early PSII assembly factor
Source: Plant Cell. 2025 Mar 17;37(6):koaf055. doi: 10.1093/plcell/koaf055 (PMC12164590; doi:10.1093/plcell/koaf055)
Supplement: koaf055_Supplementary_Data [file koaf055_supplementary_data.zip › psb28_interactive.zip!/plots/intensity/qualityPlots/peptides/EFH1_Cre02.g111700.t1.2.html]

### Cre02.g111700.t1.2

Trivial name: EFH1  
SeqCoverage [median]: 10.40  
Mass: 22.34 kDa  
  
 

| Sequence | MissedCleavage | Mass [Da] | wtIntensity | muIntensity | Score |
| --- | --- | --- | --- | --- | --- |
| EYIFNRVDQLVNADSK | 1 | 1909.95 | 0.00 | 898.58 | 34.50 |
| TAVLGY | 0 | 622.33 | 0.00 | 1755.35 | 78.00 |
| VDQLVNADSK | 0 | 1087.55 | 0.00 | 406.74 | 102.06 |
|  |  |  | 0.0 | 3060.7 |  |
